# Prenatal and accurate perinatal diagnosis of type 2 H or ductular duplicate gallbladder

**DOI:** 10.1186/s12887-018-1043-9

**Published:** 2018-02-07

**Authors:** Umberto Maggi, Giorgio Farris, Alessandra Carnevali, Irene Borzani, Paola Clerici, Massimo Agosti, Giorgio Rossi, Ernesto Leva

**Affiliations:** 1 UOC Chirurgia Pediatrica, Centro Trapianti Fegato – Fondazione IRCCS Ca’ Granda, Ospedale Maggiore Policlinico di Milano, V Francesco Sforza 35, 20121 Milan, Italy; 2 UOC Radiologia, - Fondazione IRCCS Ca’ Granda Ospedale Maggiore Policlinico di Milano, Milan, Italy; 3 Struttura neonatologia, Terapia intensiva neonatale e pediatria Verbano – Ospedale Filippo Del Ponte ASST dei Sette Laghi, Varese, Italy; 4 Department of Pathophysiology and Transplantation, University of Milan, Milan, Italy; 5 Ostetricia e Ginecologia A - Ospedale Filippo Del Ponte ASST dei Sette Laghi , Varese, Milan, Italy; 6 UO Chirurgia Epatobiliopancreatica e trapianti di fegato, Fondazione IRCCS Ca’ Granda Ospedale Maggiore Policlinico di Milano, Milan, Italy

**Keywords:** Cholangiopancreatography, Magnetic resonance, Boyden classification, Fetal sonography, Gallbladder duplication, H-type cystic duct duplication, MRCP, Gallbladder/abnormalities, Gallbladder/diagnostic imaging, Ultrasonography, Prenatal, Infant, Newborn, Pregnancy

## Abstract

**Background:**

Double gallbladder is a rare biliary anomaly. Perinatal diagnosis of the disorder has been reported in only 6 cases, and in 5 of them the diagnosis was based on ultrasound imaging only. However, the ultrasound technique alone does not provide a sufficiently precise description of cystic ducts and biliary anatomy, an information that is crucial for a correct classification and for a possible future surgery.

**Case presentation:**

At 21 weeks of gestational age of an uneventful pregnancy in a 38 year old primipara mother, a routine ultrasound screening detected a biliary anomaly in the fetus suggestive of a double gallbladder. A neonatal abdominal ultrasonography performed on postnatal day 2 confirmed the diagnosis. On day 12 the newborn underwent a Magnetic Resonance Cholangiopancreatography (MRCP) that clearly characterized the anatomy of the anomaly: both gallbladders had their own cystic duct and both had a separate insertion in the main biliary duct.

**Conclusions:**

We report a case of early prenatal suspected duplicate gallbladder that was confirmed by a neonatal precise diagnosis of a Type 2, H or ductular duplicate gallbladder, using for the first time 3D images of Magnetic resonance cholangiopancreatography in a newborn. An accurate anatomical diagnosis is mandatory in patients undergoing a possible future cholecystectomy, to avoid surgical complications or reoperations. Therefore, in case of a perinatal suspicion of a double gallbladder, neonates should undergo a Magnetic resonance cholangiopancreatography.

A review of the Literature about this variant is included.

**Electronic supplementary material:**

The online version of this article (10.1186/s12887-018-1043-9) contains supplementary material, which is available to authorized users.

## Background

Double gallbladder is a rare biliary anomaly with a reported incidence of approximately 1 in 4000 individuals [1, 2]. In adults, it is often an intraoperative finding. A perinatal diagnosis has been reported in 6 cases and in 5 of them the diagnosis was based on ultrasonography only. However, a precise characterization of cystic ducts anatomy is very important for classification and a possible future surgery. This result cannot be achieved by ultrasound examination only.

We describe a very early prenatal diagnosis of double gallbladder - at the 21st week of gestational age - confirmed soon after birth by Magnetic resonance cholangiopancreatography (MRCP) 3D images. This technique, employed for the first time in this condition, is very helpful and extremely precise. A review of the literature about this rare disorder is likewise provided [2].

## Case presentation

**Fig. 1:**
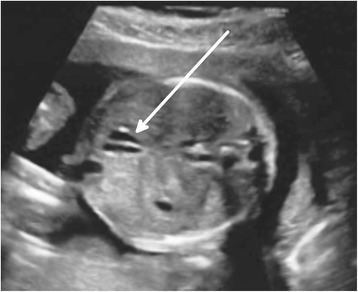
Prenatal ultrasound on the 21st week of gestational age: Transverse axial image of the fetal abdomen showing 2 adjacent fluid filled structures in the gallbladder fossa. This finding is consistent with the presence of a duplicated gallbladder

At 21 weeks of gestational age of an uneventful pregnancy of a 38 yr. old primipara mother, a routine ultrasound (US) screening of the fetus detected a biliary anomaly allegedly considered a double gallbladder (Fig. 1). Fetal biometric parameters including abdominal diameters and circumferences, liver appearance, and intrahepatic biliary system were normal.

Pregnancy was otherwise uneventful. A full-term female newborn was delivered via uncomplicated spontaneous vaginal delivery on June 18, 2016. The neonate had a birth weight of 3120 g, length of 51 cm, and normal Apgar Score (8 at 1 min and 10 at 5 min).

A neonatal abdominal US performed on postnatal day 2 confirmed the presence of two saccular structures in the gallbladder fossa, without stones and with a normal intrahepatic biliary system.

On postnatal day 4 the baby was admitted to the Unit of Pediatric Surgery of the Ospedale Mangiagalli in Milan, Italy.

**Fig. 2:**
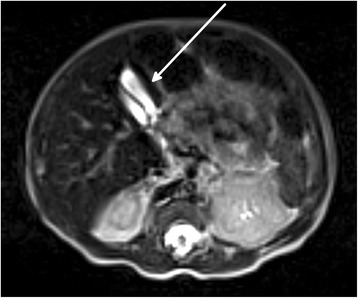
MRCP T2 hast transverse image: duplicate gallbladder

**Fig. 3:**
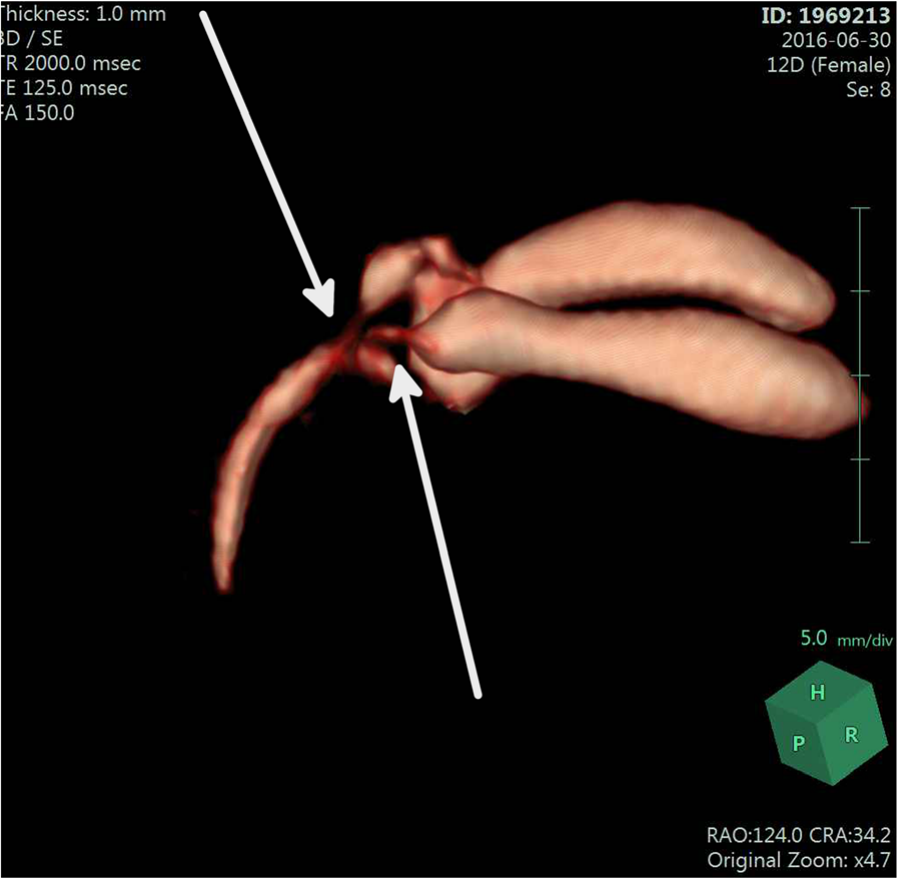
3D reconstructions images of MRCP: two gallbladders and two cystic ducts with separate insertions in the main bile duct

The patient underwent a second abdominal ultrasound that confirmed the existence of a double gallbladder. On day 12 the patient was subjected to a MRCP (Fig. 2 and Fig. 3) that precisely outlined the anatomy of the anomaly: both gallbladders had their own cystic duct and both of them had separate insertions in the main biliary duct. A diagnosis of Type 2 H or ductular duplicate gallbladder was then performed.

The patient was discharged in good health (see Additional file 1).

## Discussion and conclusion

Double gallbladder is a rare anatomic malformation of the biliary tract, arising from duplication of either 1 primordium or 2 primordia [1], because of abnormalities in embryogenesis, during the fifth and sixth weeks of gestation [3, 4]. In the first case, when the cystic primordium splits, the two gallbladders share a common cystic duct. In the second case, cystic ducts have an independent insertion in the biliary tree.

The anomaly was described for the first time in 1674 by Blasius, at autopsy of a 2-year old boy in Amsterdam. The first report of surgical removal of a double gallbladder was made by Sherren in 1911. Boyden in 1926 [5] proposed a first classification of the disorder. Another classification was introduced by Harlaftis in 1977. A left variant has also been described [6]. A classification including triple gallbladders has been recently proposed by Causey [3]. According to Harlaftis the disease incidence has an equal sex distribution.

Preoperative diagnosis is problematic [1]. Ultrasonography, the first investigation to be performed, is unable to precisely study the anatomy of the biliary tree. Computed Tomography (CT) scan, MRCP [2], and Endoscopic retrograde cholangiography (ERCP) [2] are presently the main diagnostic tools. In recent years, diagnostic apparatus has greatly improved, and, consequently, preoperative diagnosis is not unusual. However, the ultrasound imaging can only exclude or document the existance of a double gallbladder but a precise diagnosis needs to differentiate the cystic ducts with their insertion in the biliary tree [7]. According to Harlaftis suggestion, when MRCP can be performed before surgery [8] the diagnosis is very useful.

Double gallbladder can be discovered in either symptomatic [1, 4, 6, 9–11] or asymptomatic [2] adult patients [9], but a preoperative diagnosis occurs in only about 50% of cases [2, 6]. Therefore, even in the presence of a preoperative workup, only surgery with [4, 6, 9] or without intraoperative cholangiogram provides the correct diagnosis.

Prenatal or early postnatal diagnosis of double gallbladder has rarely been reported. Abdominal US of the fetus is usually performed in order to detect biliary anomalies, first of all a biliary atresia .

We found only 4 reports of prenatal or early postnatal diagnosis [7] describing 6 cases. In 5 of them, only sonography was performed. In one subject an MRCP was also performed but no 3D images are available.

In our patient, the anomaly was suspected during pregnancy as early as the 21st week of gestational age. The diagnosis was thereafter confirmed by ultrasound soon after birth although a precise anatomical definition was only obtained by MRCP. To our knowledge, the use of MRCP for this diagnosis has been reported in adults [2, 8] but only in one newborn.

Our case consists of a Type 2 H or ductular duplicate gallbladder. Identification of a dual duct as an anatomic variant is particularly important [3] as this information is useful in guiding surgical planning in view of a potential future cholecystectomy [7]. The Three-D MRCP images in the present patient, show the gallbladders and their cystic ducts with their respective insertion into the main duct. This kind of images are very detailed and have never been shown in the medical literature.

The indication for surgery for double gallbladders is uncertain in asymptomatic patients. Safioleas [9] states that surgical treatment is not required if the double gallbladder produces no symptoms [9, 10] as the incidence of gallstones is similar relative to single gallbladders.

In symptomatic patients, laparotomic [6] or laparoscopic cholecystectomy must include both gallbladders [4]; surgery must be careful to avoid reoperation [1, 2]. Indeed, failure to recognize the accessory gallbladder can result in cholecystitis in the residual gallbladder after cholecystectomy. Up to 2015 only 15 cases of laparoscopic cholecystectomy in adults for duplicate gallbladder were performed [4].

Our little patient was asymptomatic so we decided to avoid surgery.

In conclusion, six cases of prenatal duplicate gallbladder have been reported so far. Diagnosis in five of them was based on sonography only. As the type of duplicate gallbladder can be correctly characterized only if the insertions of the cystic ducts are identified, this happened only in one newborn through a neonatal MRCP .

Duplicate gallbladder is rare but we wish to emphasize that ultrasound images in the fetus or soon after birth are not sufficient. Indeed, in view of a possible surgical treatment, the precise detection of the anatomy of the biliary anomaly is mandatory. Absence of this information exposes to surgical complications or reoperations. Therefore, in case of a suspicion of diagnosis of double gallbladder, the neonate should undergo a Magnetic resonance cholangiopancreatography.

We report a case of very early prenatal suspicion of duplicate gallbladder performed with ultrasound on the 21st week of gestational age, and a neonatal very precise diagnosis with 3D images of a MRCP, never reported before, of a Type 2, H or ductular duplicate gallbladder.

## Additional file

Additional file 1: Timeline. Exact timeline of the case report from the 21st gestational week with the first suspicion of double gallbladder to the final exact diagnosis by Magnetic resonance cholangiopancreatography and discharge of the little patient. (DOC 27 kb)

## Abbreviations

CT: Computed Tomography
ERCP: Endoscopic retrograde cholangiography
MRCP: Magnetic resonance cholangiopancreatography
US: Ultrasound, Ultrasonography
