# Supplementary material for: Prenatal and accurate perinatal diagnosis of type 2 H or ductular duplicate gallbladder
Source: BMC Pediatr. 2018 Feb 7;18:38. doi: 10.1186/s12887-018-1043-9 (PMC5803916; doi:10.1186/s12887-018-1043-9)
Supplement: Additional file 1: — Timeline. Exact timeline of the case report from the 21st gestational week with the first suspicion of double gallbladder to the final exact diagnosis by Magnetic resonance cholangiopancreatography and discharge of the little patient. (DOC 27 kb) [file 12887_2018_1043_MOESM1_ESM.doc]

21 gestational age

38 years old primipara mother

routine ultrasound (US) screening of the fetus

suspicion of a double gallbladder of the fetus

June 18, 2016

Spontaneous vaginal delivery

postnatal day 2

postnatal day 12

Magnetic resonance cholangiopancreatography

Precise diagnosis of the anatomy of the anomaly

Confirmation of the suspicion of a double gallbladder of the fetus

birth weight of 3120 g, length of 51 cm, and normal Apgar Score ( 8 at 1 min and 10 at 5 min)

Neonatal abdominal US

diagnosis of Type 2 H or ductular duplicate gallbladder. Patient discharged.
